# Structural Characterization and In Vitro *α*‐Amylase Inhibitory and Bile Acid‐Binding Properties of a Glucan Isolated From Fermented Cowpea Natto

**DOI:** 10.1002/fsn3.72456

**Published:** 2026-10-12

**Authors:** Jinyi Yu, Mengyue Xu, Hui Li, Enzhao Yan, Huifang Niu, Qin Liu, Hongxing Zhang, Hongbo Wang

**Affiliations:** ^1^ School of Life Sciences Jianghan University Wuhan China; ^2^ Innovation Center for Comprehensive Utilization of Food and Medicine Homologous Specialty Resources Wuhan China; ^3^ Hubei Province Engineering Research Center for Legume Plants Wuhan China; ^4^ Wuhan (China‐Cuba) Belt and Road Joint Laboratory on Natural Products Wuhan China

**Keywords:** *α*‐Glucan, *Bacillus subtilis* natto, cowpea, glycosidic linkage, polysaccharide purification

## Abstract

Cowpea natto is a fermented legume food with potential functional properties. However, the effects of fermentation on cowpea polysaccharides remain insufficiently understood. In this study, cowpea was fermented with *Bacillus subtilis* natto, and polysaccharides extracted from unfermented cowpea (JD) and fermented cowpea natto (JD‐F) were comparatively characterized. Fermentation increased the polysaccharide yield by 35.60%. A homogeneous polysaccharide, designated JDFE1‐F, was isolated and purified from JD‐F and had a weight‐average molecular weight (*M*_w_) of 5.175 kDa. Monosaccharide composition analysis showed that JDFE1‐F was predominantly composed of glucose (Glc, 98.22%). Methylation analysis and nuclear magnetic resonance (NMR) spectroscopy revealed that JDFE1‐F possessed a backbone mainly composed of →4)‐*α*‐D‐Glc*p*‐(1→ residues, with limited substitution at the *O*‐6 position. Its branched regions contained terminal *α*‐D‐Glc*p*‐(1→ residues and minor *α*‐D‐Glc*p*‐(1 → 5)‐*α*‐L‐Ara*f*‐(1→ structural motifs. Following purification, the total carbohydrate content of JDFE1‐F increased to 85.12% ± 3.09%. JDFE1‐F exhibited an *α*‐amylase IC_50_ of 2.668 ± 0.281 mg/mL, which was significantly lower than that of JD (*p* < 0.05). At 4 mg/mL, JDFE1‐F bound 58.09% ± 2.58% of sodium glycodeoxycholate and 60.79% ± 2.87% of sodium taurodeoxycholate. The concentration required for 50% sodium glycodeoxycholate binding was significantly lower for JDFE1‐F than for JD (*p* < 0.05), whereas no significant differences among the three preparations were observed for sodium taurodeoxycholate binding. These findings indicate that fermentation with *Bacillus subtilis* natto increased the polysaccharide yield and was associated with changes in the compositional and in vitro functional characteristics of cowpea polysaccharides. The low molecular weight, glucose‐rich composition, and branched *α*‐glucan structure of JDFE1‐F may be associated with its observed in vitro activities, providing a basis for further investigation of its structure–function relationships and physiological relevance.

## Graphical abstract

Cowpea ( *Vigna unguiculata* L.) seeds were fermented with *Bacillus subtilis* natto, freeze‐dried, milled, and subjected to hot‐water extraction, protein removal, alcohol precipitation, and lyophilization to obtain the crude glucan fraction (JD‐F). JD‐F was further purified by DEAE‐Sepharose Fast Flow anion‐exchange chromatography and size‐exclusion chromatography, yielding the purified glucan JDFE1‐F for structural characterization and evaluation of its in vitro *α*‐amylase inhibitory and bile acid‐binding properties.

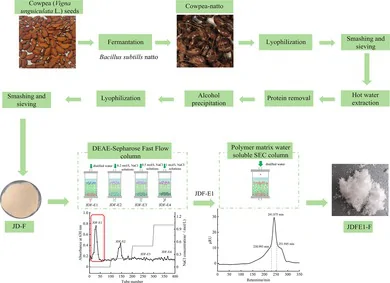

## 1 Introduction

Fermentation is an established biotechnological process that can improve the safety, nutritional quality, and shelf life of plant‐derived foods (Liu et al. 2024; Qiao et al. 2022). Natto is traditionally produced by fermenting soybean with *Bacillus subtilis* natto and contains several bioactive constituents, including nattokinase and polysaccharides. Cowpea ( *Vigna unguiculata* L.) is an important legume that is widely cultivated and consumed because of its nutritional value, environmental adaptability, and contribution to food security (Gore et al. 2025; Kim et al. 2024). It is also an important source of plant‐based proteins, carbohydrates, vitamins, and minerals for humans (Fan et al. 2023; Moyo et al. 2024). These characteristics make cowpea a potentially suitable substrate for natto‐type fermentation and the production of value‐added polysaccharide fractions.

Legume polysaccharides have attracted increasing attention because of their diverse biological activities, particularly their potential hypoglycemic and hypolipidemic effects (Bai et al. 2023; Lin et al. 2022). Wu et al. (2019) reported that cowpea polysaccharides alleviated metabolic abnormalities in diabetic mice by improving hepatic glycogen content, fasting blood glucose, triglyceride and cholesterol levels, insulin sensitivity, and pancreatic islet cell number. Consequently, the extraction, purification, structural characterization, and modification of legume polysaccharides have become important research topics (Zhu et al. 2022). However, legume polysaccharides are often recovered at relatively low yields and may contain high‐molecular‐weights or poorly soluble fractions, limiting their accessibility, functional performance, and practical application (Niyigaba et al. 2021).

Microbial fermentation offers a comparatively mild strategy for improving polysaccharide extractability and modifying naturally occurring polysaccharide structures without the chemical synthesis of new macromolecules (Wang, Zheng, Zhou, et al. 2024). During fermentation, microbial growth and enzyme secretion may disrupt plant cell‐wall matrices, release bound polysaccharides, remove or transform accompanying constituents, and alter polysaccharide molecular‐weight distributions, monosaccharide proportions, glycosidic linkages, branching patterns, and chain conformations. These changes can influence polysaccharide solubility, functional‐group accessibility, and biological activity (Wang, Zheng, Dai, et al. 2024).

Several studies have demonstrated the capacity of *Bacillus* fermentation to remodel polysaccharides. Fermentation of *Dendrobium officinale* with *Bacillus* sp. DU‐106 altered polysaccharide structure and enhanced immunostimulatory activity (Tian et al. 2019), whereas fermentation of sea cucumber polysaccharides with *Bacillus subtilis* natto improved their in vitro digestibility (Li et al. 2023). Similarly, *Bacillus subtilis* natto fermentation increased the extraction yield and water solubility of lentinan while altering its molecular weight, uronic acid content, morphology, and antioxidant activity (Xu et al. 2023). More recently, a homogeneous *α*‐glucan isolated from *Bacillus* natto‐fermented millet bran was shown to possess a predominantly →4)‐*α*‐D‐Glc*p*‐(1→ backbone with *O*‐6 branching and measurable in vitro antioxidant activity (Zhang et al. 2025). Collectively, these findings indicate that *Bacillus*‐mediated fermentation can influence the extraction, structural organization, and functional properties of plant‐derived polysaccharides.

Molecular weight can affect solubility, viscosity, molecular mobility, chain entanglement, and the accessibility of functional groups, while high‐molecular‐weight polysaccharides may exhibit a large excluded volume and strong intermolecular interactions that restrict solubility, although the relationship between molecular weight and activity is not universally linear (Wei and Zhang 2023). Monosaccharide composition and relative proportions influence the distribution of functional groups, hydrophilicity, charge characteristics, and chain conformation (Xu et al. 2024). Glycosidic linkage patterns can further regulate local and higher‐order conformations, thereby affecting molecular interactions with biological targets (Chen et al. 2024). Branching may provide additional terminal residues and accessible interaction sites, although its effects also depend on branch length, frequency, and spatial organization (Zhao et al. 2014). These structural factors may jointly determine the ability of polysaccharides to interact with digestive enzymes and bile acids. However, homogeneous polysaccharides isolated from cowpea fermented with *Bacillus subtilis* natto remain insufficiently characterized, and the potential relationships between their structural features, *α*‐amylase inhibitory activity, and bile acid‐binding capacity have not been systematically investigated.

Based on evidence that microbial fermentation can modify the physicochemical and functional properties of plant‐derived polysaccharides, we hypothesized that *Bacillus subtilis* natto fermentation could alter the polysaccharide composition of cowpea and facilitate the enrichment of fractions with distinct structural and functional characteristics. Accordingly, a homogeneous polysaccharide fraction, JDFE1‐F, was isolated and purified from fermented cowpea polysaccharides, and its molecular weight, monosaccharide composition, glycosidic linkages, backbone structure, and branching pattern were characterized using chromatographic, mass spectrometric, and nuclear magnetic resonance techniques. The chemical compositions, *α*‐amylase inhibitory activities, and bile acid‐binding capacities of JD, JD‐F, and JDFE1‐F were also comparatively evaluated to assess the potential effects of fermentation and purification on their functional properties. By integrating detailed structural characterization with comparative functional evaluation, this study provides new information on fermented cowpea polysaccharides and establishes a basis for investigating the structure–activity relationships and food‐related applications of JDFE1‐F.

## 2 Materials and Methods

### 2.1 Materials

Cowpea ( *Vigna unguiculata* L.) seeds (NO. 2) were obtained from the Hubei Province Engineering Research Center for Legume Plants (Jianghan University). *Bacillus subtilis* natto (CICC 10263) was purchased from the China Center of Industrial Culture Collection (Beijing, China; Country of Origin, Japan) and preserved at Jianghan University. The selection of this strain was predicated on its exceptional nattokinase production capacity (> 5000 FU/g under optimized conditions of 37°C and pH 7.0) and its thermostable fermentation temperature adaptability (optimal proliferation at 30°C–37°C, with > 90% enzyme retention up to 50°C), attributes that engender efficacious polysaccharide modification via nattokinase‐mediated enzymatic hydrolysis, thereby effecting a substantial diminution in molecular weight (approximately 48%) and augmentation of solubility. Luria‐Bertani (LB) liquid medium (10 g/L tryptone, 5 g/L yeast extract, 10 g/L NaCl, pH 7.0–7.5; Thermo Scientific Oxoid Co. Ltd., UK) was sterilized at 121°C for 20 min. Trifluoroacetic acid (TFA), iodomethane (CH_3_I), dimethyl sulfoxide (DMSO), 1‐phenyl‐3‐methyl‐5‐pyrazolone (PMP), sodium borodeuteride (NaBD_4_), and sodium borohydride (NaBH_4_) were purchased from Sigma Chemical Co. (St. Louis, MO, USA). All other chemicals and solvents used were of analytical grade.

### 2.2 Preparation of JD and JD‐F

#### 2.2.1 Cowpea Natto Preparation

*Bacillus subtilis* natto was transferred into LB liquid medium and incubated at 37°C for 12 h. Cowpeas were washed and soaked in purified water (*w*/*v* = 1:3) at 25°C for 16 h. Before fermentation, the samples were drained and sterilized by autoclave sterilization (0.1 MPa, 121°C) for 20 min, cooled to ambient temperature, and then incubated with 5% of the precultured inoculum (OD_600_ = 1.0 ± 0.1) in LB liquid medium. The samples underwent solid‐state fermentation in an incubator (SPX‐150B‐Z, Shanghai Boxun Co. Ltd., Shanghai, China) at 37°C for 24 h. At the end of fermentation, the cowpea natto was kept at 4°C for 24 h for after‐ripening and freeze‐dried using a vacuum freeze dryer (−85°C, 0.040 Mbar) for 18 h (Freezone 6 Plus, Labconco Corporation inc., Kansas, MO, USA) (Chu et al. 2019; Dong et al. 2020).

#### 2.2.2 Extraction of JD and JD‐F

Polysaccharides were extracted from cowpea seeds and cowpea natto by hot water extraction and named JD and JD‐F, respectively, according to Qu et al. (2024), with minor modifications. The crushed and sieved samples were degreased using 10 times the volume of ethanol at 37°C for 12 h, and the filter residue was collected and dried at 70°C. The aforementioned filter residue was extracted with distilled water using a solid/liquid ratio of 1:20 at 98°C for 6 h. Following centrifugal clarification (4320 × *g*, 10 min), the harvested supernatant underwent cold acidic precipitation using 3% TCA (1:1, *v*/*v*) at 4°C for 20 h. Following centrifugation (7760 × *g*, 10 min), the extract was harvested, and the supernatant was obtained and precipitated with three‐fold volumes of ethanol at 4°C for 20 h. Following differential centrifugation (7760 × *g*, 10 min), the pellet underwent lyophilization to yield target compounds JD and JD‐F.

### 2.3 Preparation of JDFE1‐F

JDFE1‐F was isolated and purified from JD‐F based on the methods previously described by Fan et al. and Qu et al. (Das and Sen 2024; Fan et al. 2023; Qu et al. 2024). JD‐F was purified by ion‐exchange chromatography on a DEAE‐Sepharose Fast Flow column (XK 3.0 × 50 cm) equilibrated with water. The sample was dissolved in deionized water (dH_2_O), centrifuged (7760 × *g*, 10 min), and filtered through a 0.45 μm membrane. An amount corresponding to 30% of the column volume (CV) was loaded, and stepwise elution was performed with dH_2_O, 0.2 M NaCl, 0.5 M NaCl, and 1.0 M NaCl (2 CV each) at 15 mL/min. Fractions (10 mL/tube) were collected and monitored by the anthrone–sulfuric acid assay (630 nm) and designated JD‐F (1–4). The major peak (JDF‐E1) was pooled, concentrated, dialyzed (MWCO 3.5 kDa), and lyophilized. JDF‐E1 was further purified by size‐exclusion chromatography on Sephacryl S‐300 HR (XK 2.6 × 100 cm) with isocratic elution using water at 1.5 mL/min. The corresponding fractions were pooled and lyophilized to afford JDFE1‐F.

### 2.4 Structural Characteristics

#### 2.4.1 Fourier Transform Infrared Spectroscopy (FT‐IR) Analyses

The conformation of JDFE1‐F was determined by FT‐IR spectroscopy (Nicolet iS50R, Thermo Fisher Scientific, USA). The sample was prepared by mixing the lyophilized JDFE1‐F with KBr at a 1:100 (w/w) ratio and then pressing into translucent pellets. The pellets were placed in the sample window and scanned over 4000–400 cm^−1^ with 32 scans and a resolution of 4 cm^−1^ (Li et al. 2024; Wang et al. 2018).

#### 2.4.2 Analysis of Monosaccharide Composition

JDFE1‐F's monosaccharide composition was determined via HPLC, adapting a prior method (Liu et al. 2017). JDFE1‐F (5 mg) underwent hydrolysis using 1 mL of 2 mol/L trifluoroacetic acid at 121°C for 2 h. Nitrogen stream removed solvent after thrice washing with methanol to eliminate TFA. A 0.2 mL of solution containing hydrolyzed polysaccharides (JDFE1‐F or standard monosaccharides) was derivatized with 0.2 mL of NaOH solution (0.5 mol/L) and 0.5 mL of methanol/1‐phenyl‐3‐methyl‐5‐pyrazolone (0.5 mol/L). A ZORBAX Eclipse XDB‐C18 column (Agilent Technologies, Santa Clara, CA, USA) was used. The mobile phase consisted of acetonitrile and a monopotassium phosphate buffer (0.09 mol/L, pH 6.8) in a 17:83 (v/v) ratio. The system was operated at 30°C with a steady flow rate of 0.8 mL/min. A volume of 10 μL was administered with UV analysis conducted at 250 nm for process tracking.

#### 2.4.3 Analysis of Molecular Weight

The molecular weight of JDFE1‐F was determined using high‐performance gel permeation chromatography (HPGPC) (Waters 1515, Waters, Milford, USA) as adapted from Liu et al. (2022). JDFE1‐F and 10 kinds of standard dextran (1 × 10^3^ Da, 5 × 10^3^ Da, 12 × 10^3^ Da, 25 × 10^3^ Da, 50 × 10^3^ Da, 80 × 10^3^ Da, 150 × 10^3^ Da, 270 × 10^3^ Da, 410 × 10^3^ Da and 670 × 10^3^ Da) with different molecular weights (10 mg) was combined with NaCl (0.05 mol/L) to prepare a control solution (5 mg/mL), centrifuged at 7760 × *g* for 10 min, and the clarified solution was filtered through a 0.22 μm membrane prior to analysis. A 30 μL aliquot was then injected into a tandem arrangement of water‐soluble polymer‐based SEC column (OHpak SB‐803, 804, 805 HQ; 8 × 300 mm). The system employed a 0.05 mol/L NaCl mobile phase, delivered at 0.65 mL/min to optimize separation.

#### 2.4.4 Analysis of Methylation

According to He et al. (He et al. 2024), the methylation analysis was performed this way: 1 mg of JDFE1‐F was dissolved in DMSO (1 mL). After that, it was methylated using CH_3_I (0.5 mL) in a DMSO/NaOH solution at 30°C for 2 h in darkness. Distilled water (1 mL) and dichloromethane (2 mL) were added, vortexed to mix, and centrifuged. The aqueous phase was discarded, and the washing step was repeated three times. Trifluoroacetic acid (2 mol/L, 1 mL) was added at 121°C for 2 h. Acetic acid‐methanol (2 mL, 5%, v/v) was added with nitrogen at 40°C for 2 times. Acetic anhydride (1.5 mL) was added and reacted at 100°C for 2.5 h. The substance was dissolved in dichloromethane and subjected to analyzed.

Analysis employed gas chromatography–mass spectrometry (GC–MS, 7890A‐5977B, Agilent Technologies, USA). The chromatographic conditions were as follows: an HP‐5MS capillary column (30 m × 0.25 mm × 0.25 μm, Agilent J&W Scientific, Folsom, CA, USA) was used. The injection volume was 1 μL with a split ratio of 10:1. High‐purity helium (≥ 99.999%) was employed as the carrier gas. The column temperature was initially set at 50°C and held for 1 min, then increased to 130°C at a rate of 50°C/min, further raised to 230°C at 3°C/min, and finally held steady for 2 min. The analyte's identification employed full‐scan (SCAN) mode, utilizing a verified mass spectrum (*m/z*) from 30 to 600. Inlet temperature: 230°C, four‐stage rod temperature: 150°C.

#### 2.4.5 Analysis of NMR

The details of JDFE1‐F's structure were obtained from one‐dimensional (^1^D, including ^1^H and ^13^C) and two‐dimensional (^2^D, including COSY, HSQC, HSQC‐TOCSY, HMBC, and NOESY) NMR spectra (Varian INOVA 600 MHz, Varian, US) (Liang et al. 2024; Zhao et al. 2024). The NMR testing of JDFE1‐F utilized D_2_O as the solvent. Calibration was performed using HDO with *δ* H set at 4.70 ppm and TMS with *δ* C set at 0.00 ppm.

### 2.5 Analysis of Chemical Composition

The total carbohydrate, uronic acid, and protein contents of the polysaccharides were measured using the phenol‐sulfuric acid method (DuBois et al. 1956), *m*‐hydroxydiphenyl method (Blumenkrantz and Asboe‐Hansen 1973), and Bradford method (Bradford 1976), for which glucose, galacturonic acid, and bovine albumin were used as standards, respectively.

### 2.6 *α*‐Amylase Inhibitory Activity Assay

*α*‐Amylase was selected as a primary screening target because it catalyzes the initial hydrolysis of dietary starch, and its inhibition delays glucose release and mitigates postprandial hyperglycemia. *α*‐Amylase inhibition activity by polysaccharides was assessed via a modified protocol (Gu et al. 2021; Pradhan et al. 2023). Soluble starch (1%, w/v), *α*‐amylase (0.5 mg/mL), and polysaccharide solutions (0.5, 1, 2, and 4 mg/mL) were formulated using 0.1 mol/L phosphate buffer (PBS, pH 6.8). A homogenous blend of 0.2 mL polysaccharide solution and *α*‐amylase was created and incubated (40°C, 5 min). Subsequently, 0.2 mL soluble starch was mixed (40°C, 15 min). Then, 0.4 mL of DNS reagent was introduced, and the mixture was plunged into a water bath (98°C, 4 min). The absorbance was then clocked at 540 nm. Acarbose was employed as the positive control.

$$\alpha -\text{amylase inhibitory activity}\;\left(\%\right)=\left(1-\frac{A_{2}-A_{1}}{A_{0}}\right)\times 100\%$$

where *A*_*0*_, *A*_*1*_, and *A*_*2*_ represent the absorbances of PBS (0.1 mol/L phosphate buffer, pH 6.8), the *α*‐amylase‐free sample, and the original sample, respectively.

### 2.7 Bile Acid‐Binding Capacity Assay

The methodology outlined by Qin et al. (2021) was applied to measure the bile acid‐binding capacity, incorporating slight adjustments. In 0.1 mol/L phosphate buffer solution (pH 6.3), a cocktail was created through the careful blending of polysaccharide solutions at concentrations of 0.5, 1, 2, and 4 mg/mL with pepsin (10 mg/mL), pancreatin (10 mg/mL), sodium glycodeoxycholate (0.4 mmol/L), sodium taurodeoxycholate (0.5 mmol/L). Furthermore, 0.2 mL polysaccharide extracts were treated with pepsin (0.2 mL) and HCl (0.6 mL 0.01 mol/L), digesting for 1 h at 37°C in a shaker at 150 rpm. The pH was modified to the range of 6.0 to 6.3 using 0.1 mol/L NaOH. Combine the substance with 0.8 mL pancreatin, allowing digestion (37°C, 1 h). Next, 0.8 mL of either the sodium glycodeoxycholate or taurodeoxycholate solution was administered, then reacted for 1 h at 37°C. The samples were then centrifuged at 7760 × *g* for 15 min. Combine 1 mL supernatant with 3 mL 60% H_2_SO_4_ (w/v), heat to 70°C (40 min), cool, and read absorbance at 387 nm. Under strongly acidic and heating conditions, bile acids undergo acid‐catalyzed dehydration and partial sulfonation of the steroid nucleus, yielding conjugated chromophores that display a characteristic absorption band at 387 nm. The absorbance at 387 nm is therefore proportional to the concentration of unbound (free) bile acids remaining in the supernatant after incubation with the test sample.

$$\text{bile acid}-\text{binding capacity}\;\left(\%\right)=\frac{A_{3}-A_{4}}{A_{3}}\times 100\%$$

where *A*_*3*_ represents the quantity of introduced sodium glycodeoxycholate or taurodeoxycholate (μmol), and *A*_*4*_ represents the remaining quantity (μmol).

### 2.8 Statistical Analysis

Data analysis was performed using IBM SPSS Statistics 20.0 and GraphPad Prism 9, and graphs were generated using Origin 2021. Except for structural characterization analyses, including FTIR, HPGPC, methylation analysis, and NMR spectroscopy, all quantitative data are presented as mean ± standard deviation (M ± SD) from three independent experiments (*n* = 3). The structural characterization analyses were conducted using representative samples and were not included in the statistical comparisons. Statistical differences among groups were evaluated by one‐way analysis of variance (ANOVA), followed by Duncan's multiple range test. A value of *p* < 0.05 was considered statistically significant.

## 3 Results and Discussion

### 3.1 Extraction, Isolation, and Purification of Polysaccharides

JD and JD‐F were isolated from dried cowpea seeds and cowpea natto, with extraction yields of 4.55% ± 0.34% and 6.17% ± 0.33%, respectively. The higher extraction yield of JD‐F may be associated with fermentation‐induced changes in the cowpea matrix. Previous studies have shown that *Bacillus subtilis* natto can express enzymes involved in the degradation of plant cell‐wall polysaccharides and alter the structural properties of legume‐derived substrates during fermentation (Ochiai et al. 2007; Wang, Zhang, Wei, et al. 2024). These fermentation‐induced changes may weaken the interactions between polysaccharides and other macromolecular components, thereby facilitating polysaccharide release and subsequent extraction. The extraction yields obtained in the present study were comparable to those reported previously (Chen et al. 2021; Pei et al. 2022; Qin et al. 2021). The slight variations in polysaccharide yields among studies may reflect differences in raw materials, microbial strains, fermentation conditions, and extraction procedures.

The two crude polysaccharide extracts were subsequently fractionated. JD‐F was selected for further investigation because of its relatively high polysaccharide purity, abundant uronic acid content, and low protein content, making it more suitable for detailed structural characteristics (Table 3). Fractionation of JD‐F by ion‐exchange chromatography yielded four carbohydrate‐containing fractions, namely JDF‐E1, JDF‐E2, JDF‐E3, and JDF‐E4, which were eluted with distilled water and 0.2, 0.5, and 1.0 mol/L NaCl, respectively. Based on the anthrone‐sulfuric acid elution profile and the amounts recovered after dialysis and lyophilization, JDF‐E1 and JDF‐E2 were identified as the two major carbohydrate‐containing fractions, whereas JDF‐E3 and JDF‐E4 were obtained in relatively lower amounts. Molecular‐weight analysis further indicated that JDF‐E2 consisted predominantly of oligosaccharides and other low‐molecular‐weight carbohydrate components, whereas JDF‐E1 was mainly composed of polysaccharides. Therefore, JDF‐E1 was selected for further purification by size‐exclusion chromatography. The resulting purified fraction, designated JDFE1‐F, exhibited a single symmetric peak in the HPGPC chromatogram, indicating a relatively homogeneous molecular‐weight distribution.

### 3.2 FT‐IR Analysis of JDFE1‐F

Figure 1A shows the FTIR spectrum of JDFE1‐F. The broad absorption band centered at 3405 cm^−1^ was assigned to the O‐H stretching vibration of hydrogen‐bonded hydroxyl groups, whereas the band at 2930 cm^−1^ was attributed to the C‐H stretching vibration of methylene or methine groups. These absorption features are characteristic of polysaccharides (Dai et al. 2024). The band at approximately 1640 cm^−1^ was assigned to the H‐O‐H bending vibration of bound water, suggesting hydrogen‐bonding interactions between water molecules and the hydroxyl groups of JDFE1‐F (He et al. 2024). The prominent bands at 1154, 1081, and 1020 cm^−1^ were attributed to C‐O‐C and C‐O stretching vibrations within the glycosidic linkages and pyranose rings. These signals support the presence of glucopyranose residues and are consistent with the predominance of glucose revealed by monosaccharide composition analysis (Fan et al. 2023; Yu et al. 2024). The absorption band at 855 cm^−1^ is commonly associated with an *α*‐glycosidic configuration, supporting the predominance of *α*‐linked glucopyranose residues in JDFE1‐F. Although the weak band at approximately 929 cm^−1^ has previously been associated with *β*‐glucoside structures, this signal alone is insufficient to confirm the presence of *β*‐glycosidic linkages and should be interpreted together with the methylation and NMR results (Yu et al. 2024). Overall, the FTIR spectrum confirmed the characteristic functional groups of a polysaccharide and indicated the presence of pyranose‐ring structures in JDFE1‐F. When considered together with the monosaccharide composition, methylation, and NMR results, these findings support that JDFE1‐F is a glucose‐rich polysaccharide predominantly composed of *α*‐linked glucopyranose residues, with a glucopyranose‐based main chain and a limited degree of branching. This structural pattern differs from that reported by Fan et al. (Fan et al. 2023), possibly because of differences in cowpea cultivar, fermentation treatment, extraction and purification procedures, and structural characterization methods.

**FIGURE 1:**
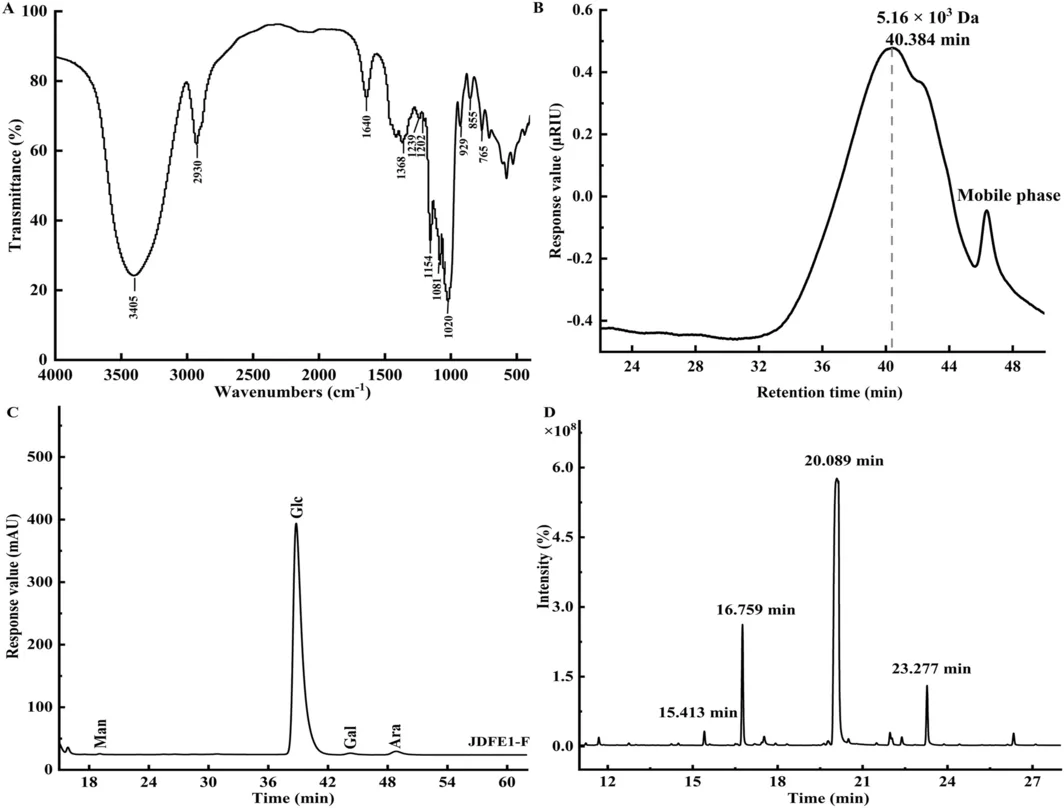
Structural feature analysis of JDFE1‐F. (A) FT‐IR spectrum. (B) HPGPC chromatograms. (C) HPLC chromatograms. (D) Total ion chromatogram.

### 3.3 Molecular Weight and Monosaccharide Composition Analysis of JDFE1‐F

As shown in Figure 1B, the HPGPC chromatogram of JDFE1‐F exhibited one predominant polysaccharide peak at a retention time of 40.384 min, corresponding to a *M*_w_ of 5.175 kDa. The minor peak observed at approximately 46.1 min corresponded to the mobile phase and was not associated with an additional polysaccharide fraction. The polydispersity index (*M*_w_/*M*_n_) of JDFE1‐F was 1.36, indicating a relatively narrow molecular‐weight distribution. As shown in Figure 1C, JDFE1‐F was predominantly composed of Glc, which accounted for more than 98% of the total monosaccharide content, with only small amounts of Man (0.024%), Gal (0.557%), and Ara (1.199%) detected. These results indicate that JDFE1‐F is a glucose‐rich polysaccharide with a relatively low molecular weight and a narrow molecular‐weight distribution (Fan et al. 2023).

### 3.4 Methylation Analysis of JDFE1‐F

Methylation results of JDFE1‐F were depicted in Figure 1D, with residue details consolidated in Table 1. JDFE1‐F consisted of four glycosidic bonds: 1,5‐Ara*f* (15.413 min), t‐Glc*p* (16.759 min), 1,4‐Glc*p* (20.089 min), and 1,4,6‐Glc*p* (23.277 min). The 1,4‐Glc*p* (81.023%) residues served as the dominant linkage units forming the main backbone of JDFE1‐F, while a smaller proportion of 1,4,6‐Glc*p* (5.575%) residues acted as branching points substituted at the *O*‐6 positions. The presence of t‐Glc*p* (12.001%) residues corresponded to terminal glucose units that cap these minor side chains, confirming that the molecule possesses a predominantly linear (1 → 4)‐linked glucan backbone with sparse *O*‐6‐linked branches. These findings align with the monosaccharide composition and FT‐IR results, further verifying that JDFE1‐F is a glucan. The consistency between the methylation‐derived linkage ratios and the monosaccharide composition indicates that the polysaccharide backbone was well preserved during methylation (Table 1).

**TABLE 1:** JDFE1‐F methylation analysis data.

Time (min)	Linkage types	Molar ratio (%)
15.413	1,5‐Ara*f*	1.400
16.759	t‐Glc*p*	12.001
20.089	1,4‐Glc*p*	81.023
23.277	1,4,6‐Glc*p*	5.575

### 3.5 NMR Spectroscopic Analysis of JDFE1‐F

As shown in Figure 2A,B, the anomeric proton and carbon signals of JDFE1‐F were distributed within the ranges of 4.48–5.24 ppm and δ 91.73–107.26, respectively. Based on the monosaccharide composition and methylation results, the major sugar residues and glycosidic linkage patterns of JDFE1‐F were assigned through the combined analysis of the one‐dimensional (^1^H and ^13^C NMR) and two‐dimensional (COSY, HSQC, HSQC‐TOCSY, HMBC, and NOESY) NMR spectra (Figure 2A–G and Table 2).

**FIGURE 2:**
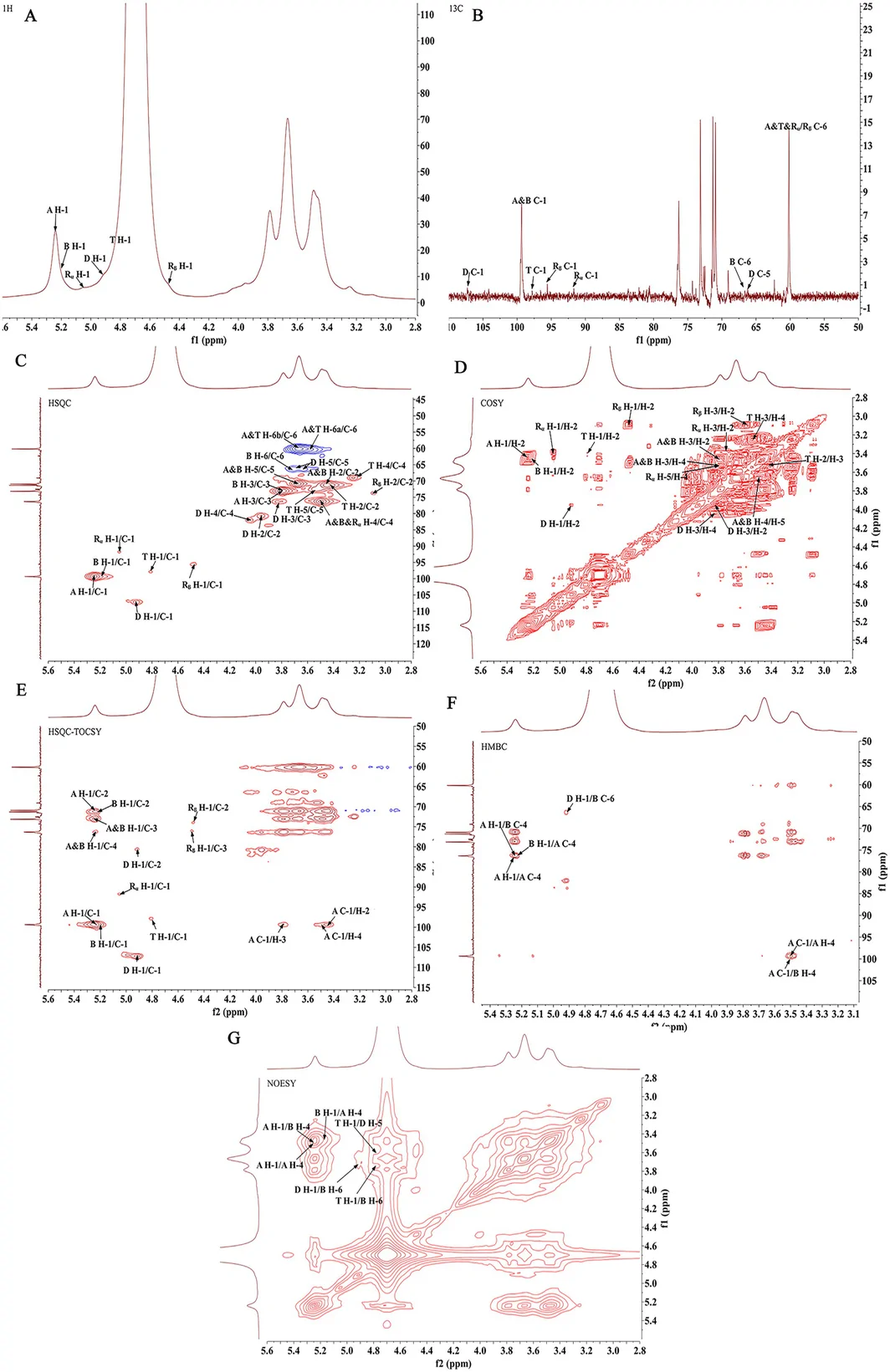
JDFE1‐F's ^1^D and ^2^D NMR spectra. (A) ^1^H NMR. (B) ^13^C NMR. (C) HSQC. (D) COSY. (E) HSQC‐TCOSY. (F) HMBC. (G) NOESY.

**TABLE 2:** ^1^H and ^13^C NMR chemical shifts of glycosyl residues of JDFE1‐F.

Glycosyl residue		Chemical shift δ (ppm)						
			1	2	3	4	5	6a/6b
A	→4)‐*α*‐D‐Glc*p*‐(1→	H	5.24	3.45	3.79	3.50	3.67	3.57/3.66
		C	99.35	71.21	73.06	76.28	71.26	60.17
B	→4,6)‐*α*‐D‐Glc*p*‐(1→	H	5.21	3.45	3.78	3.50	3.68	3.72/3.75
		C	99.32	71.21	72.72	76.29	71.21	66.70
T	*α*‐D‐Glc*p*‐(1→	H	4.80	3.42	3.51	3.25	3.56	3.57/3.66
		C	97.88	71.25	72.66	69.06	72.72	60.17
D	→5)‐*α*‐L‐Ara*f*‐(1→	H	4.91	3.95	3.83	4.04	3.62/3.71	—
		C	107.26	80.69	76.39	82.05	66.22	—
R_*α*_	→4)‐*α*‐D‐Glc*p*	H	5.05	3.38	3.73	3.50	3.79	3.57/3.66
		C	91.73	71.26	73.02	76.28	69.25	60.17
R_*β*_	→4)‐*β*‐D‐Glc*p*	H	4.48	3.09	3.57	3.62	3.45	3.55/3.74
		C	95.54	73.75	76.19	76.39	74.13	60.69

In the HSQC spectrum, a prominent anomeric cross‐peak was observed at δ 5.24/99.35 ppm (H‐1/C‐1), which falls within the characteristic chemical‐shift ranges of *α*‐Glc*p* residues, namely δ 5.22–5.32 ppm for H‐1 and δ 99–101 ppm for C‐1 (Figure 2C). This signal was therefore assigned to the anomeric H‐1/C‐1 correlation of an *α*‐Glc*p* residue (Chaves et al. 2019; Chen et al. 2023; Guo et al. 2020; He et al. 2024; Jeong et al. 2019; Leemhuis et al. 2014; Li et al. 2020; Shi et al. 2019; Wang, Li, et al. 2022; Wen et al. 2021; Wu et al. 2024). The H‐1, H‐2, H‐3, H‐4, H‐5, H‐6a, and H‐6b signals of this residue were assigned at δ 5.24, 3.45, 3.79, 3.50, 3.67, 3.57 and 3.66 ppm, respectively. The corresponding C‐1, C‐2, C‐3, C‐4, C‐5, and C‐6 signals occurred at δ 99.35, 71.21, 73.06, 76.28, 71.26, and 60.17 ppm, respectively. The downfield shifts of C‐1 and C‐4 indicated substitution at these positions, supporting the assignment of this residue as →4)‐*α*‐D‐Glc*p*‐(1→, designated residue A.

Another strong anomeric cross‐peak was observed at δ 5.21/99.32 ppm (H‐1/C‐1), close to the characteristic regions of δ 5.19–5.22 ppm for the anomeric proton and δ 99–101 ppm for the anomeric carbon of *α*‐Glc*p* residues. Combined with the methylation results and the downfield shifts of C‐4 and C‐6, this residue was assigned as →4,6)‐*α*‐D‐Glc*p*‐(1→ and designated residue B. An anomeric cross‐peak at 4.80/97.88 ppm (H‐1/C‐1) was also observed in the anomeric region of the HSQC spectrum (Figure 2C). Based on its chemical shifts and the methylation results, this signal was tentatively assigned to a terminal *α*‐D‐Glc*p*‐(1→ residue, designated residue T. JDFE1‐F also contained a small amount of a 1,5‐linked Ara*f* residue. A weak cross‐peak was detected in the anomeric region of the HSQC spectrum at approximately δ 4.90–5.00 ppm for ^1^H and δ 107–108 ppm for ^13^C. In particular, the anomeric cross‐peak at δ 4.91/107.26 ppm (H‐1/C‐1) was tentatively assigned to →5)‐*α*‐L‐Ara*f*‐(1→, designated residue D, based on previously reported chemical‐shift data (Chaves et al. 2019; Chen et al. 2023; Guo et al. 2020; Jeong et al. 2019; Li et al. 2020; Wang, Li, et al. 2022).

In the ^13^C NMR spectrum, signals at δ 91.73 ppm and δ 95.54 ppm were attributed to the anomeric carbons of reducing‐end residue. Corresponding cross‐peak were observed at δ 5.05/91.73 ppm and δ 4.48/95.54 ppm, respectively, in the anomeric region of the HSQC spectrum (Figure 2C). In addition, the COSY spectrum showed cross‐peak at δ 5.05/3.38 ppm and δ 4.48/3.09 ppm (Figure 2D), indicating that the H‐2 chemical shifts of these two reducing‐end residues were δ 3.38 ppm and 3.09 ppm, respectively. These signals were assigned to the reducing‐end *α*‐Glc*p* and *β*‐Glc*p* residues. Their C‐2 signals at δ 71.26 and 73.75 ppm were assigned to →4)‐*α*‐D‐Glc*p* and →4)‐*β*‐D‐Glc*p* reducing‐end residues, designated R_*α*_ and R_*β*_, respectively (He et al. 2024; Leemhuis et al. 2014; Shi et al. 2019).

As shown in Figure 2F,G, the inter‐residue linkage patterns of JDFE1‐F were further elucidated using the long‐range ^1^H‐^13^C correlations observed in the HMBC spectrum and the spatial ^1^H‐^1^H correlations observed in the NOESY spectrum. Several diagnostic correlations were identified. First, the HMBC cross‐peaks at δ 5.24/76.28 ppm (A H‐1/A C‐4) and δ 3.50/99.35 ppm (A H‐4/A C‐1), together with the NOESY cross‐peak at δ 5.24/3.50 ppm (A H‐1/A H‐4), supported an A‐(1 → 4)‐A linkage, corresponding to a →4)‐*α*‐D‐Glc*p*‐(1 → 4)‐*α*‐D‐Glc*p*‐(1→ sequence. Second, the HMBC correlation between A H‐1 (δ 5.24 ppm) and B C‐4 (δ 76.29 ppm), together with the NOESY cross‐peak between A H‐1 (δ 5.24 ppm) and B H‐4 (δ 3.50 ppm), supported an A‐(1 → 4)‐B linkage, indicating that a →4)‐*α*‐D‐Glc*p*‐(1→ residue was linked to the *O*‐4 position of a →4,6)‐*α*‐D‐Glc*p*‐(1→ residue. Third, the HMBC correlation between B H‐1 (δ 5.21 ppm) and A C‐4 (δ 76.28 ppm), together with the NOESY cross‐peak between B H‐1 (δ 5.21 ppm) and A H‐4 (δ 3.50 ppm), supported a B‐(1 → 4)‐A linkage, indicating that a →4,6)‐*α*‐D‐Glc*p*‐(1→ residue was linked to the *O*‐4 position of a →4)‐*α*‐D‐Glc*p*‐(1→ residue. Fourth, the NOESY cross‐peak between T H‐1 (δ 4.80 ppm) and B H‐6 (δ 3.72/3.75 ppm) supported a T‐(1 → 6)‐B linkage, suggesting that a terminal *α*‐D‐Glc*p*‐(1→ residue was attached to the *O*‐6 position of a →4,6)‐*α*‐D‐Glc*p*‐(1→ residue. Fifth, the HMBC correlation between D H‐1 (δ 4.91 ppm) and B C‐6 (δ 66.70 ppm), together with the NOESY cross‐peak between D H‐1 (δ 4.91 ppm) and B H‐6 (δ 3.72/3.75 ppm), supported a D‐(1 → 6)‐B linkage, indicating that a →5)‐*α*‐L‐Ara*f*‐(1→ residue was attached to the *O*‐6 position of a →4,6)‐*α*‐D‐Glc*p*‐(1→ residue. Finally, the NOESY cross‐peak between T H‐1 (δ 4.80 ppm) and D H‐5 (δ 3.62/3.71 ppm) supported a T‐(1 → 5)‐D linkage, corresponding to an *α*‐D‐Glc*p*‐(1 → 5)‐*α*‐L‐Ara*f*‐(1→ structural motif. Collectively, these HMBC and NOESY correlations indicated that the backbone of JDFE1‐F mainly contained consecutive →4)‐*α*‐D‐Glc*p*‐(1→ and →4,6)‐*α*‐D‐Glc*p*‐(1→ residues linked through *O*‐4. The *O*‐6 position of the →4,6)‐*α*‐D‐Glc*p*‐(1→ residues could be substituted by either terminal *α*‐D‐Glc*p*‐(1→ residues or →5)‐*α*‐L‐Ara*f*‐(1→ residues, with the latter further bearing terminal *α*‐D‐Glc*p*‐(1→ residues at *O*‐5.

Methylation analysis identified 1,4‐Glc*p*, t‐Glc*p*, 1,4,6‐Glc*p*, and 1,5‐Ara*f*, with molar proportions revealed a molar ratio of 81.023%, 12.001%, 5.575%, and 1.400%, respectively. When normalized to t‐Glc*p*, their approximate molar ratio was 6.75:1.00:0.46:0.12. This suggested that JDFE1‐F was primarily made up of →4)‐*α*‐D‐Glc*p*‐(1→. Based on the results of monosaccharide composition, methylation analysis results, and NMR information analysis, the preliminary structure of JDFE1‐F was deduced to be a →4)‐*α*‐D‐Glc*p*‐(1→ main chain glucan, with side chains at the *O*‐6 position of a small amount of *α*‐D‐Glc*p*. A single side chain configuration, comprising structure *α*‐D‐Glc*p*‐(1→ and minor *α*‐D‐Glc*p*‐(1 → 5)‐*α*‐L‐Ara*f*‐(1→ segments, constitutes a proposed structural model for JDFE1‐F, depicted in Figure 3.

**FIGURE 3:**
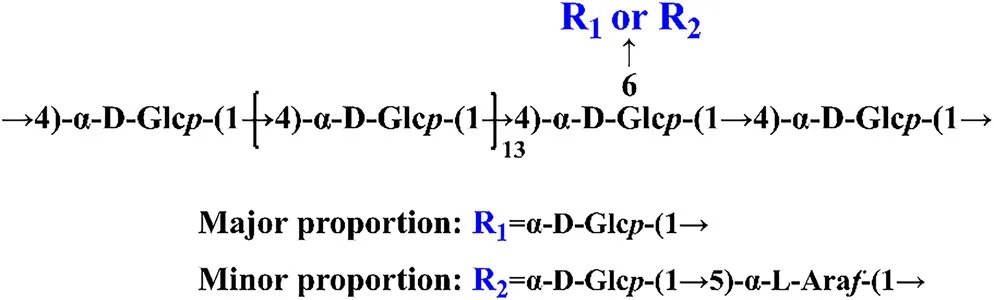
Illustration of the potential repeating structure in JDFE1‐F.

### 3.6 Chemical Composition of JD, JD‐F, and JDFE1‐F

As shown in Table 3, the chemical components of JD, JD‐F, and JDFE1‐F were determined. JD contained 33.58% ± 2.82% total carbohydrate, 8.47% ± 0.43% protein, and 1.01% ± 0.49% uronic acid. After fermentation and crude polysaccharide extraction, the total carbohydrate and uronic acid contents of JD‐F increased to 44.03% ± 2.15% and 8.22% ± 1.54%, respectively, whereas protein was below the detection limit. The absence of detectable protein in JD‐F may reflect differences in the extractable protein fraction after fermentation together with TCA‐mediated deproteinization during crude polysaccharide preparation. Fermentation may also have disrupted the cowpea matrix and enhanced the release or extractability of carbohydrate‐containing components. Comparable alterations in plant matrix structure and carbohydrate bioconversion following *Bacillus subtilis* fermentation have been reported in fermented soybean meal (Wang, Zhang, Wei, et al. 2024). Therefore, the increased relative abundance of carbohydrates in JD‐F may reflect both fermentation‐associated improvements in carbohydrate extractability and the removal of non‐carbohydrate constituents during extraction.

**TABLE 3:** Chemical composition of JD, JD‐F, and JDFE1‐F.

Sample	JD	JD‐F	JDFE1‐F
Total carbohydrate (%)	33.58 ± 2.82^c^	44.03 ± 2.15^b^	85.12 ± 3.09^a^
Uronic acid (%)	1.01 ± 0.49^b^	8.22 ± 1.54^a^	9.16 ± 0.77^a^
Protein (%)	8.47 ± 0.43^a^	ND	ND

*Note:* Data are presented as the mean ± SD from three independent experiments (*n* = 3). Different lowercase superscript letters within the same column indicate significant differences among samples (*p* < 0.05). ‘ND’ indicates no detected.

Following purification, the total carbohydrate content of JDFE1‐F increased further to 85.12% ± 3.09%, whereas its uronic acid content was 9.16% ± 0.77%, and protein remained below the detection limit. The substantial increase in total carbohydrate content indicates effective enrichment of the polysaccharide fraction and removal of residual non‐polysaccharide constituents. By contrast, the comparatively modest change in uronic acid content between JD‐F and JDFE1‐F suggests that purification predominantly enriched the overall polysaccharide fraction without strongly selecting for uronic‐acid‐rich components. The continued absence of detectable protein was also consistent with the combined effects of deproteinization and subsequent purification.

In contrast, Chen et al. (Chen et al. 2022) reported a decrease in the polysaccharide content of whole soybeans following fermentation with *Bacillus subtilis* natto. This finding does not necessarily conflict with the increased carbohydrate proportion observed in JD‐F because the two studies examined different analytical materials. Chen et al. quantified polysaccharides directly in the whole fermented substrate, whereas the present study determined the relative chemical composition of an isolated crude polysaccharide fraction after extraction and deproteinization. Differences in legume species, microbial strains, fermentation conditions, analytical methods, and extraction or purification procedures may further account for the variation among studies. Collectively, these findings indicate that the carbohydrate enrichment observed in JD‐F and JDFE1‐F should be interpreted primarily in the context of the specific fermentation, extraction, and purification procedures used in the present study.

### 3.7 *α*‐Amylase Inhibitory Activity of JD, JD‐F, and JDFE1‐F in Vitro

As shown in Figure 4A, JD, JD‐F, and JDFE1‐F inhibited *α*‐amylase in a concentration‐dependent manner. At a sample concentration of 0.5 mg/mL, the inhibition rates of all three samples exceeded 20%. At 4 mg/mL, the inhibition rates of JD, JD‐F, and JDFE1‐F reached 44.43% ± 1.17%, 50.80% ± 0.78%, and 52.55% ± 1.37%, respectively. As summarized in Table 4, the IC_50_ values of JD‐F and JDFE1‐F were 3.509 ± 0.311 and 2.668 ± 0.281 mg/mL, respectively, both significantly lower than that of JD 7.286 ± 0.992 mg/mL (*p* < 0.05). JDFE1‐F showed the lowest numerical IC_50_ value, indicating that fermentation was associated with enhanced *α*‐amylase inhibition and that subsequent purification enriched a fraction with comparatively strong activity.

**FIGURE 4:**
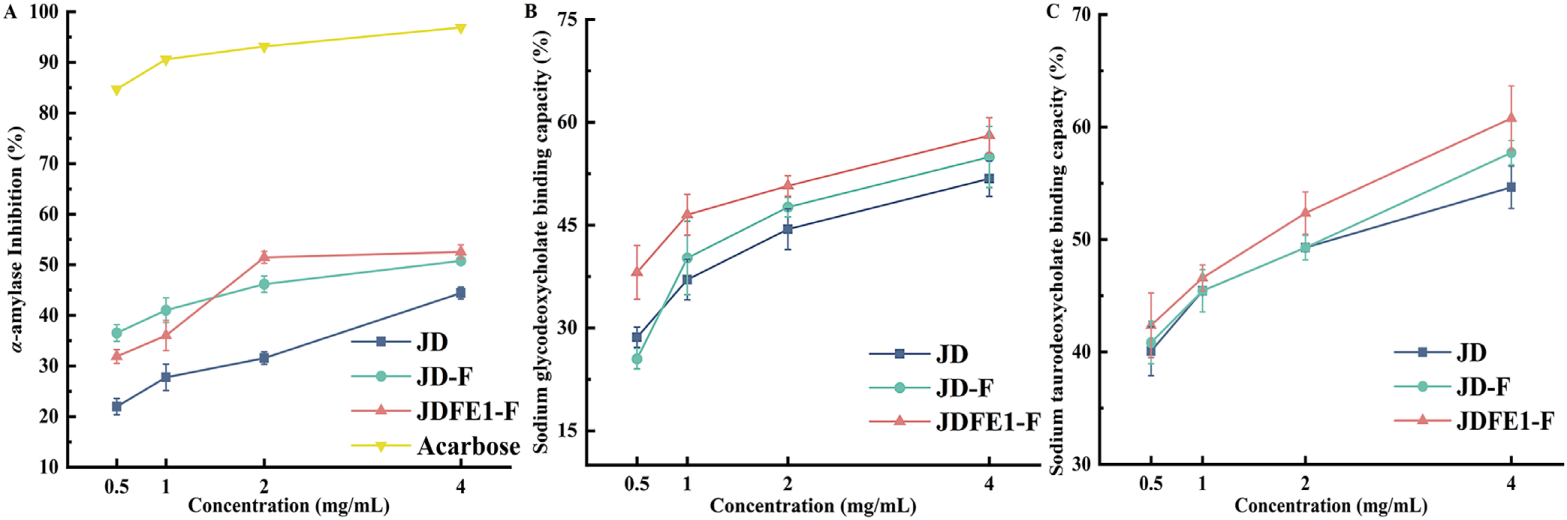
*α*‐Amylase inhibitory activity and bile acid‐binding capacity of JD, JD‐F, and JDFE1‐F. (A) *α*‐Amylase inhibition. (B) Sodium glycodeoxycholate‐binding capacity. (C) Sodium taurodeoxycholate‐binding capacity. Data are presented as the mean ± SD from three independent experiments (*n* = 3).

**TABLE 4:** IC_50_ values for *α‐*amylase inhibition and concentrations required to achieve 50% bile acid binding by JD, JD‐F, and JDFE1‐F.

Sample	*α‐*Amylase inhibition IC_50_ (mg/mL)	Concentration required for 50% sodium glycodeoxycholate binding (mg/mL)	Concentration required for 50% sodium taurodeoxycholate binding (mg/mL)
JD	7.286 ± 0.992^a^	3.431 ± 0.898^a^	2.133 ± 0.638^a^
JD‐F	3.509 ± 0.311^b^	2.602 ± 0.648^ab^	1.759 ± 0.348^a^
JDFE1‐F	2.668 ± 0.281^b^	1.711 ± 0.574^b^	1.338 ± 0.458^a^

*Note:* Data are presented as the mean ± SD from three independent experiments (*n* = 3). IC_50_ represents the concentration required to inhibit 50% of *α*‐amylase activity. The concentration required for 50% bile acid binding represents the sample concentration at which the absolute bile acid‐binding rate reached 50%, as determined from the concentration‐response curve. Different lowercase superscript letters within the same column indicate significant differences among samples (*p* < 0.05).

The stronger activity of JD‐F relative to JD may reflect fermentation‐induced changes in polysaccharide extractability and molecular structure. Extracellular enzymes produced by *Bacillus subtilis* natto may partially remodel plant cell‐wall components and polysaccharide chains, thereby altering molecular‐weight distribution, monosaccharide proportions, branching, and functional‐group accessibility. Fermentation‐induced changes in molecular weight and glycosidic linkages have been associated with altered functional properties of cereal polysaccharides (Tsafrakidou et al. 2020), while fermented blue honeysuckle polysaccharides exhibited stronger *α*‐amylase inhibition than their unfermented counterparts (Pei et al. 2022). A recent study similarly found that fermentation increased the carbohydrate proportion, altered monosaccharide ratios, reduced molecular weight, and enhanced the antioxidant activity of Chinese yam polysaccharides (Wang et al. 2025).

The numerically lower IC_50_ value of JDFE1‐F than that of JD‐F may partly reflect its higher polysaccharide purity. Purification increased the total carbohydrate content from 44.03% ± 2.15% in JD‐F to 85.12% ± 3.09% in JDFE1‐F. Thus, at the same sample concentration, JDFE1‐F contained a greater effective amount of polysaccharide and fewer non‐polysaccharide constituents that might dilute or interfere with the measured response. Purification may therefore increase the apparent mass‐normalized activity by enriching active polysaccharides, although the removal of synergistic co‐extracted compounds may also alter the response. Accordingly, purity affects the measured activity but does not necessarily determine the intrinsic activity of the polysaccharide itself. This interpretation is consistent with evidence that fermentation‐related improvements in polysaccharide activity commonly coincide with changes in purity and multiple structural properties (Wang, Zheng, Dai, et al. 2024; Wang et al. 2025).

Monosaccharide composition and molecular weight may further influence the activity of JDFE1‐F. The type and relative proportion of monosaccharides determine the distribution of functional groups, charge characteristics, hydrophilicity, branching potential, and chain conformation, thereby affecting interactions with biological targets (Wang, Zheng, Lai, et al. 2024). JDFE1‐F is a glucose‐rich *α*‐glucan composed predominantly of →4)‐*α*‐D‐Glc*p*‐(1→ residues with limited *O*‐6 branching. Its glucopyranose residues provide abundant hydroxyl groups that may participate in hydrogen bonding and other noncovalent interactions with *α*‐amylase. However, glucose abundance alone cannot explain the activity because hydroxyl accessibility also depends on linkage pattern, branching, and conformation. Molecular weight likewise regulates solubility, diffusion, chain flexibility, steric hindrance, and functional‐group accessibility, and polysaccharides generally require an appropriate rather than universally low molecular weight to exert optimal activity (Wang et al. 2023). The relatively low molecular weight of JDFE1‐F (5.175 kDa) may favor its dispersion and molecular contact with *α*‐amylase but is unlikely to support the formation of an extensive viscous or macromolecular barrier around starch granules.

Polysaccharides may inhibit *α*‐amylase through direct enzyme binding, adsorption onto starch, or restriction of enzyme‐substrate contact (Deng et al. 2020), with molecular weight, monosaccharide composition, branching, and chain conformation collectively regulating these processes (Zhang et al. 2024). Hydroxyl‐rich glucomannans have been reported to interact with *α*‐amylase through hydrogen bonding, whereas their high‐molecular‐weight counterparts may additionally form diffusion barriers (Li et al. 2026). JDFE1‐F is structurally distinct from these high‐molecular‐weight polymers. Although a low uronic acid content was detected, uronic acid monosaccharides were not detected in the monosaccharide composition analysis, indicating that uronic acid components represent only a minor contribution to JDFE1‐F. Therefore, a carboxyl‐mediated mechanism is unlikely to be the major basis for its *α*‐amylase inhibitory activity. Moreover, given its relatively low molecular weight, a viscosity‐mediated diffusion‐barrier mechanism similar to that proposed for high‐molecular‐weight glucomannans is also unlikely to be the principal explanation. Given its glucose‐rich composition and predominantly *α*‐(1 → 4)‐linked glucan structure, JDFE1‐F may instead interact with the carbohydrate‐binding subsites of *α*‐amylase through substrate‐like recognition and hydrogen bonding, thereby interfering with enzyme‐substrate association. However, because *α*‐(1 → 4)‐linked glucans may also serve as substrates for *α*‐amylase, the present results cannot distinguish direct enzyme inhibition from alternative‐substrate competition or related assay‐dependent effects. Therefore, direct active‐site occupancy and a specific kinetic inhibition type cannot be assigned based on the present data. Overall, the *α*‐amylase inhibitory activity of JDFE1‐F likely reflects the combined effects of polysaccharide enrichment, molecular weight, monosaccharide composition, linkage pattern, branching, and functional‐group accessibility, rather than any single structural determinant.

### 3.8 Bile Acid‐Binding Capacity of JD, JD‐F, and JDFE1‐F in Vitro

As shown in Figure 4B,C, JD, JD‐F, and JDFE1‐F bound more than 50% of both bile acids at a sample concentration of 4 mg/mL. At this concentration, JDFE1‐F bound 58.09% ± 2.58% of sodium glycodeoxycholate and 60.79% ± 2.87% of sodium taurodeoxycholate. As summarized in Table 4, the concentration required for 50% sodium glycodeoxycholate binding was 1.711 ± 0.574 mg/mL, which was significantly lower than that of JD 3.431 ± 0.898 mg/mL (*p* < 0.05), whereas JDFE1‐F and JD‐F did not differ significantly (*p* > 0.05). No significant differences among the three preparations were detected for sodium taurodeoxycholate binding (*p* > 0.05). Thus, the statistically supported improvement was restricted to the binding of sodium glycodeoxycholate by JDFE1‐F relative to JD, rather than representing a general enhancement toward both bile acids.

Fermentation may affect bile acid binding by altering polysaccharide extractability, molecular‐weight distribution, monosaccharide proportions, chain conformation, and the accessibility of interaction sites. Structural remodeling during microbial fermentation has previously been associated with changes in the bile acid‐binding capacity of plant polysaccharides (Bian et al. 2022; Pang et al. 2021). Fermentation of Chinese yam polysaccharides was also reported to increase carbohydrate content, modify monosaccharide proportions, reduce molecular weight, and enhance bioactivity (Wang et al. 2025).

The total carbohydrate content increased from 44.03% ± 2.15% in JD‐F to 85.12% ± 3.09% in JDFE1‐F, indicating substantial enrichment of the polysaccharide fraction. When activity is expressed per unit mass of sample, such enrichment increases the effective amount of polysaccharide available for bile acid interaction and reduces potential interference from proteins, pigments, phenolics, or other co‐extracted constituents. Nevertheless, total carbohydrate content does not establish molecular homogeneity, and purification may remove either interfering compounds or components that act synergistically with polysaccharides. The absence of significant differences between JD‐F and JDFE1‐F in the binding of either bile acid indicates that polysaccharide enrichment alone was insufficient to consistently enhance the activity. Instead, the measured binding probably reflects the combined effects of polysaccharide content and structural properties (Wang, Zheng, Dai, et al. 2024; Wang et al. 2025).

Molecular weight can influence bile acid binding through its effects on solubility, molecular mobility, viscosity, chain entanglement, and surface accessibility. Lower‐molecular‐weight polysaccharides may diffuse more readily and expose interaction sites, whereas larger chains may provide multivalent contacts, greater viscosity, or physical entrapment of bile acids. Consequently, the relationship between molecular weight and bioactivity is context‐dependent rather than uniformly inverse (Gao et al. 2017; Hu et al. 2013; Kim and White 2010; Wang et al. 2023). The relatively low molecular weight of JDFE1‐F (5.175 kDa) may facilitate its dispersion and molecular contact with sodium glycodeoxycholate. However, it is unlikely to produce extensive viscosity‐mediated entrapment, and the present data do not establish molecular weight as the principal determinant of its activity.

Monosaccharide composition may further regulate bile acid binding by defining the distribution of hydroxyl groups, charge characteristics, branching pattern, hydrophilicity, and chain conformation (Wang, Zheng, Lai, et al. 2024). JDFE1‐F is a glucose‐rich *α*‐glucan containing more than 98% Glc and only 0.557% Gal. Therefore, its bile acid‐binding behavior is unlikely to be primarily attributable to Gal residues. Instead, its predominantly →4)‐*α*‐D‐Glc*p*‐(1→ structure, limited *O*‐6 branching, and abundant hydroxyl groups may collectively influence its interactions with bile acid. These hydroxyl groups may participate in hydrogen bonding, although their accessibility and spatial arrangement also depend on linkage pattern, branching, and higher‐order conformation. The strong binding reported for galactomannans should therefore be regarded as a structure‐specific observation and not as evidence that Gal universally enhances bile acid binding (Behera et al. 2023).

Previous molecular‐interaction studies suggest that polysaccharides can associate with bile acids through hydrogen bonding, hydrophobic interactions, surface adsorption, and physical sequestration (Wang, Xiong, Li, et al. 2024; Wang, Yang, et al. 2022). For JDFE1‐F, hydrogen bonding involving accessible hydroxyl groups and surface‐level association appears more plausible than mechanisms dependent on galactose enrichment or extensive macromolecular entrapment. Differences in conjugated groups, charge distribution, steric configuration, and hydrophobicity of sodium glycodeoxycholate and sodium taurodeoxycholate may result in different affinities for the same *α*‐glucan structure. Such bile acid specificity may explain why JDFE1‐F showed greater sodium glycodeoxycholate‐binding capacity than JD, whereas its binding capacity did not differ significantly from JD‐F, and no significant improvement was observed for sodium taurodeoxycholate.

Overall, JDFE1‐F exhibited greater sodium glycodeoxycholate‐binding capacity than JD, whereas its sodium taurodeoxycholate‐binding capacity was not significantly different from those of JD and JD‐F. Its bile acid‐binding behavior should therefore be interpreted in relation to its glucose‐rich *α*‐glucan structure rather than its negligible galactose content. The observed activity likely reflects the combined effects of polysaccharide enrichment, molecular weight, glucosidic‐linkage pattern, limited branching, chain conformation, and hydroxyl‐group accessibility, rather than any single compositional or structural feature.

## 4 Conclusion

A homogeneous polysaccharide fraction, JDFE1‐F, with a *M*_*w*_ of 5.175 kDa was isolated and purified from fermented cowpea polysaccharides. Structural analyses showed that JDFE1‐F was a glucose‐rich *α*‐glucan containing 98.22% Glc and small amounts of Man, Gal, and Ara. Its backbone was composed predominantly of →4)‐*α*‐D‐Glc*p*‐(1→residues with limited *O*‐6 substitution, while its branches mainly contained terminal *α*‐D‐Glc*p*‐(1→ residues and minor *α*‐D‐Glc*p*‐(1 → 5)‐*α*‐L‐Ara*f*‐(1→ units. Following purification, JDFE1‐F showed a substantially higher total carbohydrate content than JD‐F. JDFE1‐F also exhibited appreciable in vitro *α*‐amylase inhibitory and bile acid‐binding activities. Its *α*‐amylase IC_50_ was significantly lower than that of JD, while its sodium glycodeoxycholate‐binding capacity was significantly greater than that of JD. However, no significant differences among JD, JD‐F, and JDFE1‐F were observed for sodium taurodeoxycholate binding. These results indicate that fermentation followed by purification yielded a low‐molecular‐weight, glucose‐rich *α*‐glucan fraction with distinct structural and in vitro functional characteristics. More broadly, this study links the detailed structural features of a fermented cowpea polysaccharide fraction with its in vitro functional performance, thereby extending current knowledge of legume‐derived polysaccharides and providing a basis for their further evaluation as functional food ingredients. Several limitations should nevertheless be acknowledged. Because no specific de‐starching treatment was applied, a contribution from starch‐derived *α*‐glucans to JDFE1‐F cannot be completely excluded. In addition, the structural changes occurring during fermentation were not fully resolved, and the *α*‐amylase inhibitory and bile acid‐binding activities were evaluated only in vitro. Future studies should further clarify the structural origin, structure–activity relationships, and physiological relevance of JDFE1‐F through comparative structural analysis, mechanistic studies, simulated digestion, and in vivo validation.

## Author Contributions

**Jinyi Yu:** writing – original draft, software, methodology, formal analysis, data curation. **Mengyue Xu:** methodology, investigation, writing – original draft, formal analysis, project administration, data curation. **Hui Li:** conceptualization, writing – review and editing, supervision, resources. **Enzhao Yan:** writing – review and editing, validation, data curation. **Huifang Niu:** writing – review and editing, validation, data curation. **Qin Liu:** methodology, formal analysis, data curation. **Hongxing Zhang:** writing – review and editing, data curation. **Hongbo Wang:** writing – review and editing, supervision, resources, funding acquisition, conceptualization.

## Funding

This research was supported by Wuhan Municipal University Industry‐University‐Research Project, grant number (CXY202202).

## Conflicts of Interest

The authors declare no conflicts of interest.

## Data Availability

The data that support the findings of this study are available from the corresponding author upon reasonable request.
